# Effects of impaired steryl ester biosynthesis on tomato growth and developmental processes

**DOI:** 10.3389/fpls.2022.984100

**Published:** 2022-09-29

**Authors:** Alma Burciaga-Monge, Joan Manel López-Tubau, Natalie Laibach, Cuiyun Deng, Albert Ferrer, Teresa Altabella

**Affiliations:** ^1^ Plant Synthetic Biology and Metabolic Engineering Program, Centre for Research in Agricultural Genomics (CRAG), CSIC-IRTA-UAB-UB, Barcelona, Spain; ^2^ Department of Biochemistry and Physiology, Faculty of Pharmacy and Food Sciences, Universitat de Barcelona, Barcelona, Spain; ^3^ Department of Biology, Healthcare and the Environment, Faculty of Pharmacy and Food Sciences, Universitat de Barcelona, Barcelona, Spain

**Keywords:** CRISPR-Cas9, germination, lipid droplets, phytosterols, senescence, sterol acyltransferase, steryl ester, tomato

## Abstract

Steryl esters (SE) are stored in cytoplasmic lipid droplets and serve as a reservoir of sterols that helps to maintain free sterols (FS) homeostasis in cell membranes throughout plant growth and development, and provides the FS needed to meet the high demand of these key plasma membrane components during rapid plant organ growth and expansion. SE are also involved in the recycling of sterols and fatty acids released from membranes during plant tissues senescence. SE are synthesized by sterol acyltransferases, which catalyze the transfer of long-chain fatty acid groups to the hydroxyl group at C3 position of FS. Depending on the donor substrate, these enzymes are called acyl-CoA:sterol acyltransferases (ASAT), when the substrate is a long-chain acyl-CoA, and phospholipid:sterol acyltransferases (PSAT), which use a phospholipid as a donor substrate. We have recently identified and preliminary characterized the tomato (*Solanum lycopersicum* cv. Micro-Tom) SlASAT1 and SlPSAT1 enzymes. To gain further insight into the biological role of these enzymes and SE biosynthesis in tomato, we generated and characterized CRISPR/Cas9 single knock-out mutants lacking SlPSAT1 (*slpsat1*) and SlASAT1 (*slasat1*), as well as the double mutant *slpsat1* x *slasat1*. Analysis of FS and SE profiles in seeds and leaves of the single and double mutants revealed a strong depletion of SE in *slpsat1*, that was even more pronounced in the *slpsat1* x *slasat1* mutant, while an increase of SE levels was observed in *slasat1*. Moreover, *SlPSAT1* and *SlASAT1* inactivation affected in different ways several important cellular and physiological processes, like leaf lipid bo1dies formation, seed germination speed, leaf senescence, and the plant size. Altogether, our results indicate that SlPSAT1 has a predominant role in tomato SE biosynthesis while SlASAT1 would mainly regulate the flux of the sterol pathway. It is also worth to mention that some of the metabolic and physiological responses in the tomato mutants lacking functional SlPSAT1 or SlASAT1 are different from those previously reported in Arabidopsis, being remarkable the synergistic effect of SlASAT1 inactivation in the absence of a functional SlPSAT1 on the early germination and premature senescence phenotypes.

## Introduction

Sterols are essential structural and functional components of most eukaryotic cell membranes and, as such, they play an important role in several physiological processes (Wollam and Antebi, 2011; Zhang et al., 2020). Cholesterol and ergosterol are the sole end products of sterol biosynthesis in animals and fungi, respectively. In plants, however, over 250 different sterol species, also named phytosterols, have been identified. Although their amounts and relative proportions vary between plant species and tissues, the most abundant sterols are usually β-sitosterol and stigmasterol, the end products of the 24-ethylsterols branch, and campesterol, the final product of the 24-methylsterols branch. Some members of the Solanaceae, Liliaceae, and Scrophulariaceae families accumulate also significant amounts of cholesterol (Hartmann, 2004).

In addition to their role as structural components of cell membranes, and independently of their function as precursors of brassinosteroid hormones (BR), phytosterols can also act as signaling molecules that regulate plant growth and development. Mutants defective in genes encoding enzymes of the post-squalene segment of the phytosterol pathway, such as *dry2*/*sqe1*, *smt1*, *smt2*, *cpi1*, *cyp51a*, *hyd1*, and *hyd2*/*fackel*, show developmentally impaired phenotypes (Jang et al., 2000; Peng et al., 2002; Clouse, 2002; Posé et al., 2009; Carland et al., 2010), and specific inhibitors of sterol biosynthetic enzymes suppress cell growth (Yates et al., 1991; Wentzinger et al., 2002; Gas-Pascual et al., 2015; Shimada et al., 2019). But not only sterol deficiency has deleterious effects on plant growth and development, an excess of sterols is also harmful for plants, although the mechanisms underlying the toxic effects of sterol overaccumulation in plant cells are still poorly understood. It has been recently suggested that excess sterols might inhibit cellular activity of vegetative organs by inducing the expression of genes related to biotic and abiotic stress (Shimada et al., 2020). Cell membranes with enhanced levels of sterols may lose the ability to retain water and leak iron, thus resulting in iron deficiency (Shimada et al., 2020). Interestingly, depletion of membrane sterols also leads to misregulation of genes related to biotic and abiotic stress responses and alters iron homeostasis (Manzano et al., 2016). Altogether, these and other observations support the well-established view that membrane sterol levels must be tightly controlled to avoid the negative effects of perturbed sterol homeostasis on cell viability and overall plant performance.

Regulation of free sterols (FS) levels in the membrane can be achieved by modulating the carbon flux of the sterol biosynthesis pathway and/or by converting excess FS into steryl esters (SE), which are a non-toxic form of storage sterols in the cytoplasm. Very recently, HISE1 (HIGH STEROL ESTER 1) has been identified as a key regulatory factor in Arabidopsis sterol homeostasis (Shimada et al., 2019). It has been proposed that HISE1 suppresses excess sterol formation by downregulating the protein levels of HMG-CoA reductase, the major rate-limiting enzyme of the sterol pathway whose activity is tightly controlled at different levels from transcriptional to post translational (Wentzinger et al., 2002; Nieto et al., 2009; Leivar et al., 2011; Doblas et al., 2013; Robertlee et al., 2017). In the event that excess sterols are still present, they can be acylated to form SE, which are subsequently segregated and stored in cytoplasmic lipid droplets (LD). The buffering effect of SE formation is especially evident when sterol biosynthesis is enhanced, since in these conditions FS levels remain essentially unchanged while excess sterols accumulate as SE in LDs (Maillot-Vernier et al., 1991; Gondet et al., 1994; Wilkinson et al., 1994; Schaller et al., 1995; Bouvier-Navé et al., 2010; Shimada et al., 2019). These intracellular structures are present in the seed and leaf cells of many land plants, and their function and content may differ depending on the plant tissue and species. Thus, seed LDs function as storage compartments for lipids, while in leaves they seem to act as subcellular factories with a role in senescence and fungal infection (Mckegney et al., 1995; Bouvier-Navé et al., 2010; Shimada et al., 2014; Shimada et al., 2015). Regarding the neutral lipid composition of these vesicular-structures, although TAGs are the major component in most seed LDs (Tzen et al., 1993), some of them, like those from canola, lotus or maize, are enriched in SE (Kemp and Mercer, 1968; Hernández-Pinzón et al., 1999; Harker et al., 2003), while leaf LDs accumulate mainly SE or wax esters (Parker and Murphy, 1981). LDs are dynamic organelle which size and number can be modulated by genetic or environmental factors (Tatenaka et al., 2019).

The proposed model for maintaining membrane sterol homeostasis (Shimada et al., 2019) highlights a central role of sterol esterification in this process (Lewis et al., 1987; Dyas and Goad, 1993; Chang et al., 1997; Sturley, 1997; Schaller, 2004). SE formation implies the esterification of a long-chain fatty acid group to the free hydroxyl at C3 position of the FS four-ring backbone. This reaction is catalyzed by a family of enzymes known as sterol acyltransferases, which can be classified into acyl-CoA:sterol acyltransferases (ASAT; EC 2.3.1.26) and phospholipid:sterol acyltransferases (PSAT; EC 2.3.1.43) depending on whether the acyl donor substrate is a long-chain fatty acyl-CoA or a phospholipid, respectively (Korber et al., 2017). The sterol moiety of SE includes the bulk sterol species present in the FS fraction and also many other less abundant sterols and biosynthetic intermediates, the latter being in some cases the predominant sterols in the SE fraction (Ferrer et al., 2017). Different fatty acids may also be found esterified at the C3 position, covering a wide range of lengths (from C12 to C22), but the most common are palmitic, stearic, oleic, linoleic and linolenic acids (Dyas and Goad, 1993). The structural diversity of plants SE reflects the relevance of the esterification process and their specialized functions in different tissues and organs. Thus, SE are especially abundant in anther tapetal cells, pollen grains, seeds and the phloem (Dyas and Goad, 1993; Hernández-Pinzón et al., 1999; Wu et al., 1999; Harker et al., 2003; Behmer et al., 2013; Villette et al., 2015). High levels of SE are also accumulated in senescent leaves (Bouvier-Navé et al., 2010), in mutant plants overproducing sterols (Gondet et al., 1994; Schaller et al., 1995; Shimada et al., 2020), and when the flux through the sterol pathway is forced to increase by supplying cell cultures with pathway intermediates (Wilkinson et al.,1994). Thus, the existing data indicate that SE serve as a reversible storage pool of sterols that helps to maintain the proper levels of FSs in cell membranes during normal plant growth and development, thus preventing the potential destabilizing effect that changes of FS levels above or below a certain threshold may have on membrane structure and function. As this is likewise important under special circumstances, FS homeostasis is crucial in stress conditions (Kumar et al., 2018), during senescence and aging (Bouvier-Navé et al., 2010) and also when free sterols are required immediately in important quantities, for instance during seed germination and early seedling development and pollen tube growth (Dyas and Goad, 1993). To meet these demands, it is necessary to achieve the correct balance between SE formation and mobilization, two biochemical processes that are still poorly understood in plants.

Plant sterol acyltransferase activity has been reported to be primarily associated with membrane fractions in different species (Garcia and Mudd, 1978; Zimowski and Wojciechowski, 1981a; Zimowski and Wojciechowski, 1981b; Kalinowska et al., 1989; Bouvier-Navé and Benveniste, 1995; Banas et al., 2005; Chen et al., 2007), but so far only ASAT and PSAT enzymes from Arabidopsis and tomato have been cloned and functionally characterized (Banas et al., 2005; Chen et al., 2007; Lara et al. 2018). In Arabidopsis, the characterization of T-DNA insertion mutants defective in the expression of *PSAT1* and *ASAT1* genes revealed that PSAT1 plays a major role in the synthesis of SE in seeds and in the maintenance of leaf viability during aging, since a premature senescence phenotype associated to decreased SE levels was observed in leaves of the *psat1* mutant lines, but not in the *asat1* ones (Bouvier-Navé et al., 2010). More recently, it has been reported that the Arabidopsis *hise1* mutant shows normal growth phenotype and similar sterol content than WT leaves, because excess sterols are esterified by *PSAT1* and stored in LDs (Shimada et al., 2019). However, the *hise1-3 psat1-2* double mutant exhibited a severe growth phenotype due to high sterol content in the leaves, in which the presence of LDs was not detected, while these structures were present in WT and *hise1* mutant leaves (Shimada et al., 2019). Thus, PSAT1 plays a critical role in the suppression of sterol overaccumulation through their esterification and subsequent storage in LDs. In a previous work, we identified, cloned and characterized the PSAT1 and ASAT1 enzymes of tomato (Lara et al., 2018). This allowed us to undertake the study reported here, in which we have generated and characterized tomato (cv. Micro-Tom) CRISPR-Cas9-edited loss-of-function mutans lacking PSAT1 and ASAT1. The results obtained contribute to deepen the current understanding on the role of SE and the enzymes involved in their synthesis in tomato plant growth and development.

## Materials and methods

### Generation of CRISPR/Cas9 vectors and plant transformation

**Figure 1:**
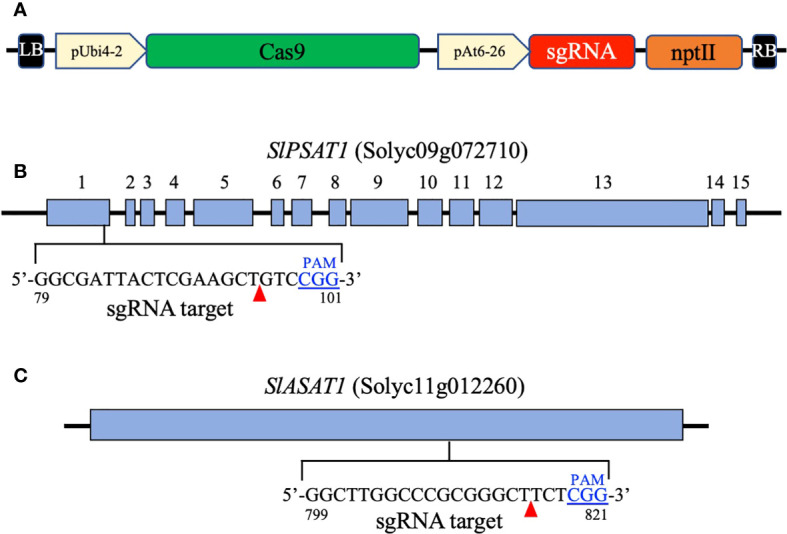
Map of the T-DNA region in the binary vectors pDE-Cas9 PSAT1 and pDE-Cas9 ASAT1 **(A)**. It includes the parsley *ubiquitin 4-2* gene promoter (*pUbi4-2*), the Arabidopsis *U6 snRNA-26* gene promoter (*pAt6-26*), the sequence coding for the Streptococcus pyogenes CAS9 nuclease (*Cas9*), the single guide RNA sequences targeting the *SlPSAT1* and *SlASAT1* genes (sgRNA), and the kanamycin resistance gene (*nptII*). Exons of the *SlPSAT1* gene **(B)** and the coding region of the *SlASAT1* gene **(C)** are represented by blue boxes. The sgRNA target sequences in the *SlPSAT1* and *SlASAT1* genes are also shown. The protospacer adjacent motif (PAM) is shown in blue and predicted CAS9 nuclease cleavage sites are indicated by red arrowheads.

Recombinant plasmids for CRISPR/Cas9-mediated genome editing were generated as described by Schiml et al. (2016) using a modified version of the pDE-Cas9 plasmid (Karlsruhe Institute of Technology), in which the phosphinothricin resistance gene (BAR) was replaced by the kanamycin resistance gene (NPTII) by restriction digest with *Hind*III and subsequent ligation, yielding the plasmid pDE-Cas9-Kan^R^. The sgRNAs targeting *SlPSAT1* (Solyc09g072710) and *SlASAT1* (Solyc11g012260) were designed using the on-line Breaking-Cas tool (http://bioinfogp.cnb.csic.es/tools/breakingcas/) (Oliveros et al., 2016). The oligonucleotide pairs encoding the selected sgRNAs (**Supplementary Table S1**) were annealed and cloned into the *Bbs*I site of the pEn-Chimera entry vector (Addgene). The resulting pEn-Chimera/ASAT1 and pEn-Chimera/ASAT1 plasmids were sequenced to exclude cloning artifacts. Plasmids pEn-Chimera/ASAT1 and pEn-Chimera/PSAT1 were recombined with pDE-Cas9-Kan^R^ destination vector yielding plasmids pDE-Cas9 ASAT1 and pDE-Cas9 PSAT1 (**Figure 1**), which were transferred to the *A. tumefaciens* strain GV3101::pMP90 prior to transform tomato (cv. Micro-Tom) cotyledons (Fernandez et al., 2009). The presence of the transgenes in kanamycin-resistant tomato plants (T0) was checked by PCR amplification of a 442-bp fragment of the *AtU6-26* promoter using primer pairs shown in **Supplementary Table 1** and leaf genomic DNA isolated with the cetyltrimethyl ammonium bromide (CTAB) method (Richards et al., 1994).

### Mutation analysis in CRISPR/Cas9-edited *slpsat1* and *slasat1* mutants

Genomic DNA fragments of 303 and 293 bp spanning the *SlASAT1* and *SlPSAT1* target sites, respectively, were amplified by PCR using DNA from transgenic plants as template, AccuPrime™ Taq DNA Polymerase High Fidelity (Thermo Fisher Scientific), and specific primer pairs flanking the target sites (**Supplementary Table S1**). The PCR products from T0 transgenic plants were cloned into pGEM^®^-T Easy (Promega) and the inserts of at least 10 independent clones were Sanger sequenced and analyzed with Serial Cloner version 2.6.1 (Perez, 2004). In subsequent generations, the bulk PCR products were directly Sanger sequenced after purification with the Gel and PCR clean-up kit (Macherey-Nagel) using primers sgRNA ASAT1 Fw and sgRNA PSAT1 Fw (**Supplementary Table 1**). The resulting sequence trace files were analyzed with the Tracking of Indels by Decomposition (TIDE) software (http://tide.nki.nl) (Brinkman et al., 2014).

### Generation of *slpsat1* x *slasat1* double mutant

To generate the *slpsat1* x *slasat1* double knock-out mutant, sepals, petals and the anther cone of unopened flower buds of homozygous *slasat34* plants were removed with forceps to expose the stigma and style, and all open flowers near the emasculated flowers were also removed to minimize the risk of self-fertilization. Pollen was collected from open flowers of homozygous *slpsat31* plants, and pollination of *slasat34* flowers was performed 24 h after emasculation by dipping the exposed stigma into the pollen (Chetelat and Peacock, 2013). Seeds from the resulting fruits were collected and double heterozygous mutant plants were initially selected by PCR-based genotyping of the two mutations as described above. Additional generations were obtained through self-pollination until *slpsat31* x *slasat34* plants carrying both mutant alleles in homozygosis were identified as described above.

### RT-qPCR expression analysis

For RT-qPCR gene expression analysis, cDNA samples were prepared from leaf DNA-free RNA obtained using the Maxwell RSC Plant RNA Kit with the Maxwell RSC Instruments (Promega) according to manufacturer’s instructions. Real-time PCR assays were performed in triplicate with SYBR Green I Master (Roche Diagnostics) using a Light Cycler 480 detection system (Roche Diagnostics) as described in Ramirez-Estrada et al. (2017). Specific primer pairs for target genes *SlPSAT1*, *SlASAT1* and *SlSAG12* (Solyc02g076910), and the actin gene (Solyc03g078400) used as reference gene, are shown in **Supplementary Table 1**.

### Imaging lipid droplets

Lipid droplets were stained with TopFluor^®^ cholesterol essentially as described in Zhou et al. (2019). Leaf discs of about 0.5 cm in diameter were excised from fresh leaves, transferred into a 20 mL syringe containing 2 mL 10 μM TopFluor Cholesterol (Avanti Polar Lipids) and 30 μM methyl-β-ciclodextrin (Fischer Scientific), and infiltrated under negative pressure by pulling the plunger for 30s. After the atmospheric pressure was recovered, leaf discs were kept in the staining solution for an additional 4 h in darkness. LD images were acquired by confocal scanning fluorescence microscopy using a Olympus FV1000 microscope and the FV10-ASW software (Olympus) in Z-stack mode. TopFluor^®^ cholesterol was excited at 488 nm and fluorescence was detected at 490-520 nm. LD were counted from each image using ImageJ 1.48v software and averaged by leaf area.

### Aging of detached leaves

Leaflets of fully green second and third leaves detached from 7 week-old-plants were placed in distilled water (20 mL) in Petri dishes and stored in the dark at 24°C for ten days. Leaf images were taken with a Nikon Z50 camera. The non-necrosed area of senescing leaves was measured as pixels by image processing. Necrosed vs non necrosed leaf surface was measured using Fiji (https://imagej.net/software/fiji/) image processing package of ImageJ2.

### Chlorophyll measurements

Chlorophyll was measured as described by Porra (2002). Briefly, total chlorophyll from fresh leaves (250 mg) was extracted using aqueous 80% acetone. After centrifugation at 13,000 x g for 2 min, the chlorophyll was quantified spectrophotometrically in the supernatant as described in Zhang et al. (2009).

### Sterol analysis

Free and esterified sterols were quantified by GC-MS in extracts from tomato seeds (200-400 mg) and the third and fourth leaves (3 g) as described in Lara et al. (2018). Normalization of the SE levels to the FS levels to determine the esterified fraction of sterols (EF) was calculated as 100*SE/(SE+FS).

## Results

### Generation of tomato *slpsat1* and *slasat1* knock-out mutants

**Figure 2:**
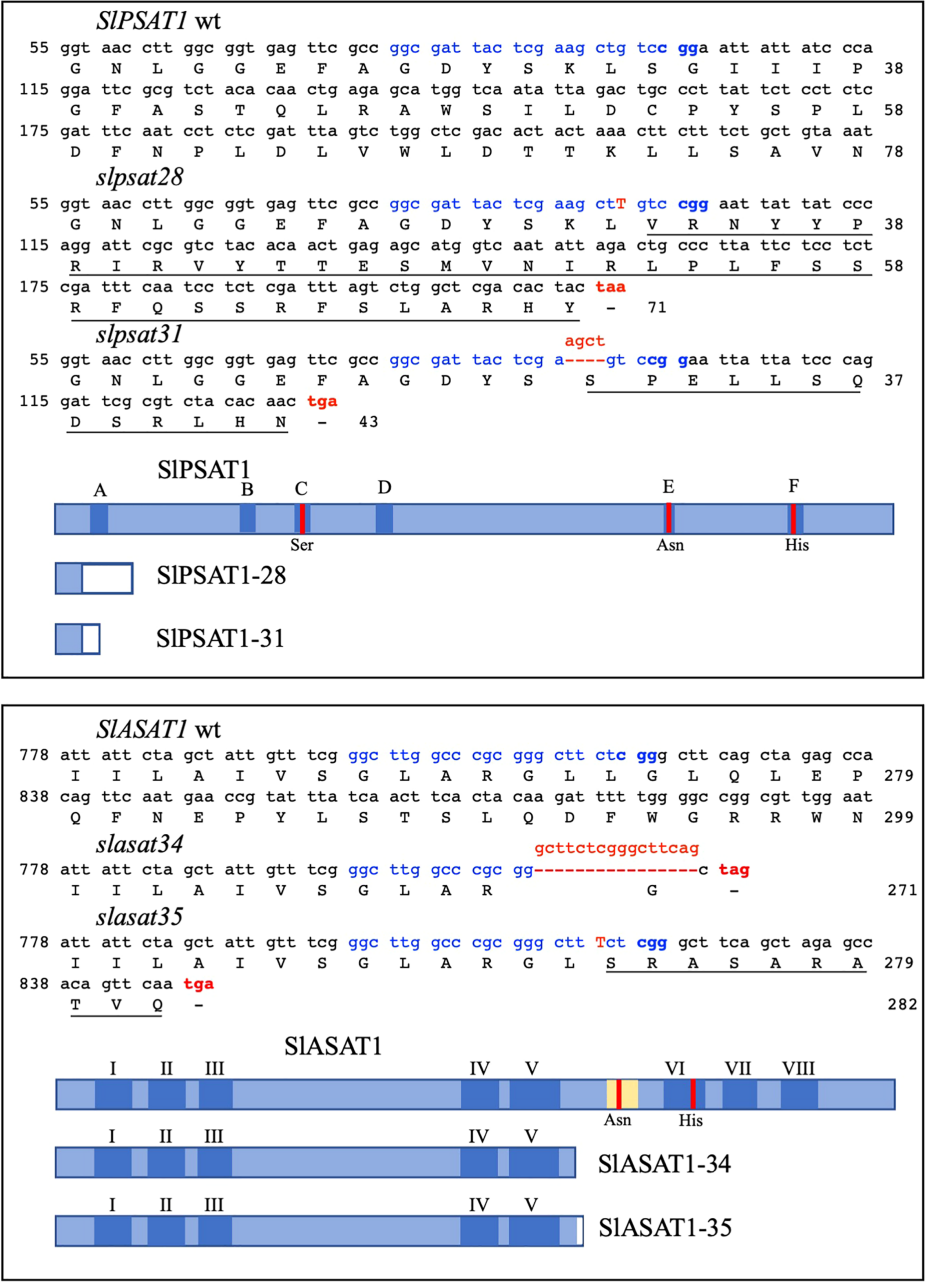
CRISPR/Cas9-induced knock-out mutations in the *SlPSAT1* and *SlASAT1* genes. Nucleotide sequences surrounding the sgRNA target site in *SlPSAT1* (upper box) and *SlASAT1* genes (lower box), and the resulting mutated sequences after imperfect NHEJ repair are shown. The sgRNA target sequences are depicted in blue, including the PAM motif highlighted in bold. The single nucleotide insertions in *slpsat28* and *slasat34* mutant alleles are shown in red capital letters, while deletions of 4 and 16 nucleotides in *slpsat31* and *slasat35* alleles, respectively, are shown in red above the dashed lines. Premature stop codons as a result of frameshift mutations are shown in red. Nucleotides are numbered on the left. The amino acids encoded by the wt and mutant alleles are shown below the respective nucleotide sequences and are numbered on the right. Amino acids in the mutant proteins that are different from those in the wild type protein sequences are underlined. The wild type SlPSAT1 and SlASAT1 proteins as well as their corresponding truncated versions are shown by blue boxes. Dark blue boxes represent the six conserved domains A-F found in LCAT proteins, and the eight predicted transmembrane sequences (I – VIII) of SlASAT1. The amino acid residues (Ser, Asn and His) of the catalytic triad in regions C, E, and F of SlPSAT1, and the SlASAT1 catalytic amino acid residues (Asn and His) are indicated by red vertical lines. The acyl-CoA binding site in SlASAT1 is shown in yellow. White boxes in the truncated proteins represent amino acid sequences not found in the wild type proteins arising from the frameshift mutations introduced by the NHEJ repair mechanism.

To investigate the biological role of SlASAT1 and SlPSAT1, tomato (cv. Micro-Tom) plants were transformed with CRISPR/Cas9 cassettes (**Figure 1**) designed to target the nucleotide sequences coding for the amino acid sequences GLARGLLG (positions 267 to 274 in SlASAT1) and GDYSKLSG (positions 27 to 34 in SlPSAT1). Primary transformants (T0) were first screened for the presence of the CRISPR/Cas9 constructs by PCR amplification of the *AtU6-26* promoter that drives the expression of the sgRNAs. Next, genomic fragments encompassing the corresponding sgRNA target sites were amplified by PCR, cloned and sequenced. Comparison of the obtained sequences with those of the equivalent genomic regions of WT plants revealed the presence of a variety of short indels ranging from 1 to 16 nucleotides in 14 *slasat1* and 7 *slpsat1* T0 mutant plants. A similar analysis conducted in the respective T1 and T2 progenies allowed the identification of plant lines with CRISPR/Cas9-induced homozygous mutations in the *SlPSAT1* and *SlASAT1* genes, among which, mutant lines *slpsat28*, *slpsat31*, *slasat34* and *slasat35* were selected for further characterization. Lines *slpsat28* and *slasat34* showed single nucleotide insertions at the predicted Cas9 editing sites, while lines *slpsat31* and *slasat35* had deletions of 4 and 16 nucleotides, respectively (**Figure 2**). All these frameshift mutations were expected to result in truncated forms of both enzymes predicted to be catalytically inactive. The native SlPSAT1 protein consists of 630 amino acid residues, in sharp contrast to the predicted sequences of 43 (*slpsat28*) and 71 (*slpsat31*) amino acid residues of proteins encoded by the *slpsat1* mutant alleles. In the case of SlASAT1, the WT enzyme consists of 444 amino acids, while the truncated proteins encoded by the mutant alleles are predicted to have 271 (*slasat34*) and 282 (*slasat35*) amino acids, thus lacking the key catalytic amino acid residues, Asn299 and His343, found in all MBOATs (Hofmann, 2000; Huang et al., 2014).

### Effect of *SlPSAT1* and *SlASAT1* inactivation on the profile of free and esterified sterols in tomato seeds and leaves

**Figure 3:**
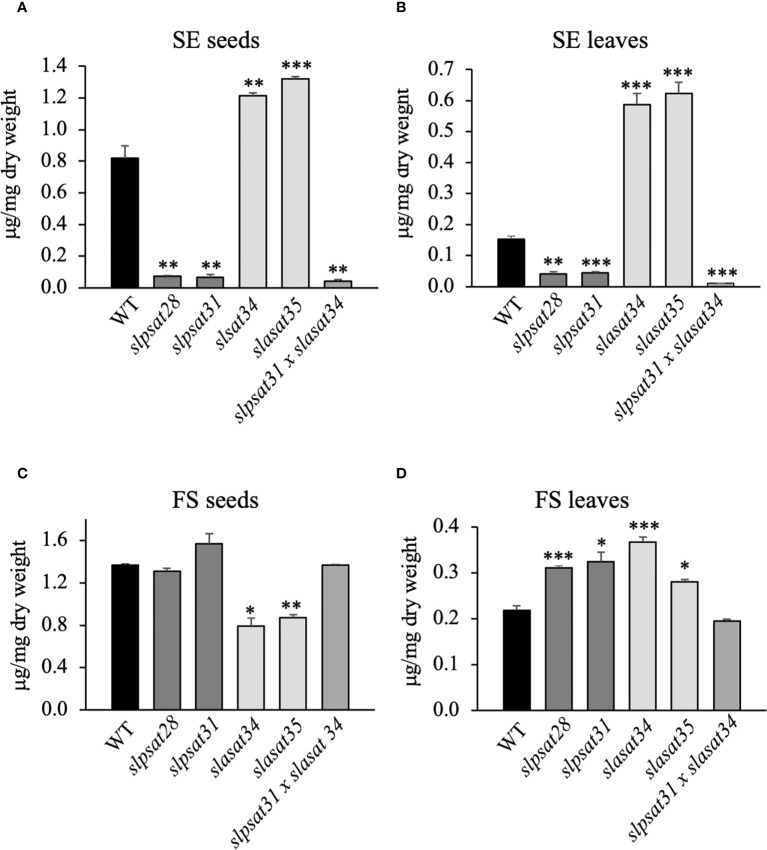
Total levels of free and esterified sterols in seeds and leaves of *slpsat1*, *slasat1*, and *slpsat1 x slasat1* mutans. Quantification of steryl esters **(A, C)** and free sterols **(B, D)** was carried out in seeds and the third and fourth leaf of one-month-old plants. Data are presented as mean ± SEM from three (seeds) and four (leaves) biological replicates per genotype. Asterisks indicate values that are significantly different compared to those in the control plants determined by t-test (*P<0.05; **P<0.01; ***P<0.005).

The impact of *SlPSAT1* and *SlASAT1* inactivation on free and esterified sterol levels was investigated in seeds and leaves of the *slpsat1* and *slasat1* mutant plants. *SlPSAT1* inactivation led to a drastic depletion of total SE levels in the seeds of both *slpsat1* lines to less than 10% (8.9% in *slpsat28* and 8.2% in *slasat31*) compared to those in WT seeds (**Figure 3A** and **Supplementary Table 2**). A significant but less severe SE reduction was also observed in the leaves, as SE levels were reduced below 30% (26.7% in *slpsat28* and 29% in *slasat31*) compared to WT leaves (**Figure 3B** and **Supplementary Table 3**). Rather unexpectedly, the levels of SE in seeds and leaves of both *slasat1* mutants increased instead of decreasing. Indeed, the total SE content in the *slasat1* seeds increased by about 50% (48% in *slasat34* and 61% in *slasat35*) compared to WT seeds (**Figure 3A** and **Supplementary Table 2**), while a much stronger effect was observed in the leaves, which showed approximately a three-fold increase in SE levels (284% in *slasat34* and 308% in *slasat35*) compared to WT leaves (**Figure 3B** and **Supplementary Table 3**). Taken together, these results demonstrated that loss of function of *SlPSAT1* and *SlASAT1* has the same qualitative effects on the SE levels in seeds and leaves, although there are some quantitative differences. On the contrary, the effects of impaired sterol esterification on total FS levels were clearly different in both organs. Thus, while *SlPSAT1* inactivation had no significant impact on seeds FS content and *SlASAT1* knock-out caused a reduction in FS levels of about 60% (58% in *slasat34* and 63% in *slasat35*) those in WT seeds (**Figure 3C** and **Supplementary Table 2**), the leaves of *slpsat1* and *slasat1* mutants showed a moderate but significant enhancement of FS levels ranging from 1.3- to 1.7-fold the WT levels (**Figure 3D** and **Supplementary Table 3**). All these changes were reflected on the total EF values (**Table 1**), which were clearly lower in both *slpsat1* seeds (5.3% and 4.1% in *slpsat28* and *slpsat31*, respectively) and leaves (11.6% and 12% in *slpsat28* and *slpsat31*, respectively) than in the corresponding WT organs (37.4% and 41% in seeds and leaves, respectively), but markedly increased in *slasat1* seeds (about 60% in both mutant lines) and leaves (61.5% and 68.9% in *slasat34* and *slasat35*, respectively). These data reinforce the contrasting effects of *SlPSAT1* and *SlASAT1* inactivation on the total SE content in tomato seeds and leaves. A detailed analysis of SE and FS profiling in both organs showed that inactivation of both tomato sterol acyltransferases affected to a greater or lesser extend the contents of all measured free and esterified sterol species in seeds (**Supplementary Table 2**) and leaves (**Supplementary Table 3**), which resulted in a strong reduction of the EF of all the analyzed species in seeds and leaves of *slpsat1* mutants compared to WT (**Table 1**). In fact, only esterified β-sitosterol could be detected in seeds of both mutant lines, with 5.5- and 6.5-fold reductions of the EF in *slpsat28* and *slpsat31*, respectively, while in leaves, esterified β-sitosterol and cholesterol were detected with reductions of their EF ranging from 2.5- to 4.5-fold in both *slpsat1* mutants. In the *slasat1* mutants the higher values of the total EF compared to the WT were primarily due to an increase of the stigmasterol EF in seeds (2- and 2.8-fold in *slasat34* and *slasat35*, respectively) and leaves (more than 3-fold in both *slasat1* mutants), while increases ranging from 1.5- and 1.9-fold were observed in the EF of other sterol species, with the exception of cholesterol in seeds and campesterol in leaves, which were only slightly enhanced or remained unchanged, respectively (**Table 1**). Overall, these results demonstrated that SlPSAT1 has a predominant role in SE biosynthesis while SlASAT1 would mainly have a regulatory role of the sterol biosynthesis pathway.

**Table 1:** Esterified sterol fractions in seeds and leaves of wt, *slasat1, slpsat1* and *slpsat1 x slasat1* mutants.

Esterified sterol fraction (%)							
SEEDS		WT	*slasat34*	*slasat35*	*slpsat28*	*slpsat31*	*slpsat31 x slasat34*
**Total sterols**		**37.4**	**60.5**	**60.2**	**5.3**	**4.1**	**3.0**
Cholesterol		76.3	89.9	87.0	11.1	n.d.	16.7
Campesterol		40.3	62.8	46.9	n.d.	n.d.	n.d.
Stigmasterol		13.9	38.4	26.7	0.3	n.d.	2.7
Sitosterol		28.0	45.5	53.2	4.3	5.1	2.2
Isofucosterol		50.3	77.3	75.2	n.d.	n.d.	n.d.
**LEAVES**		**WT**	***slasat34***	***slasat35***	***slpsat28***	***slpsat31***	***slpsat31 x slasat34***
**Total sterols**		**41.2**	**61.5**	**68.9**	**11.6**	**12.0**	**5.2**
Cholesterol		39.7	65.8	64.8	16.5	12.0	n.d.
Campesterol		51.4	52.1	51.8	n.d.	n.d.	n.d.
Stigmasterol		7.4	26.7	23.1	n.d.	n.d.	n.d.
Sitosterol	35.5		59.8	64.8	7.8	10.1	17.3

Values were calculated according to 100*SE/(SE+FS). n.d. stands for not detected. Values shown in bold correspond to the esterified fraction of the total sterols.

**Figure 4:**
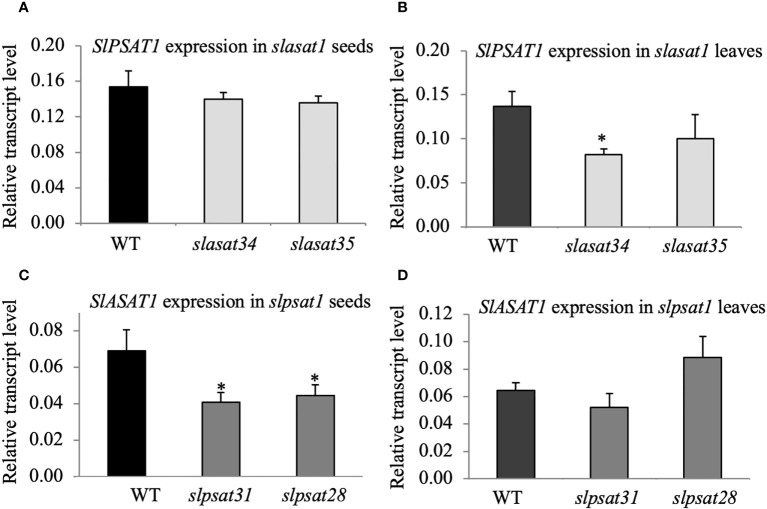
SlPSAT1 and SlASAT1 mRNA levels in seeds and leaves of *slasat1* and *slpsat1* mutants. RT-qPCR analyses were performed using RNA samples obtained from the same tissue samples used for sterol analysis (seeds and the third and fourth leaf of one-month-old WT, *slpsat1* and *slasat1* plants). The mRNA levels of the *actin* gene were used to normalize transcript levels of *SlPSAT1* **(A, B)** and *SlASAT1* **(C, D)**. Data are presented as mean ± SEM from two biological replicates per genotype with three technical replicates each (n=6). Asterisks indicate values that are significantly different compared to those in the WT plants determined by t-test (*P ≤ 0.05).

To investigate the possible existence of transcriptional crosstalk between the two sterol acyltransferases, we measured the expression of the *SlPSAT1* and *SlASAT1* genes in the *slasat1* and *slpsat1* mutants, respectively. A significant decrease (1.6- to 1.7-fold) of *SlASAT1* expression was observed in *slpsat1* seeds (**Figure 4C**) but not in leaves (**Figure 4D**), while the *SlPSAT1* expression in *slasat1* seeds was similar to WT (**Figure 4A**) and a significant decrease (1.7-fold) was observed in *slasat34* leaves (**Figure 4B**). The lack of effect of *SlASAT1* inactivation on *SlPSAT1* expression in seeds and the opposite trend of changes in *SlPSAT1* expression (decreased) and SE levels (enhanced) in leaves, indicate that the increased levels of SE in the *slasat1* mutants cannot be attributed to a transcriptional up-regulatory response of the intact *SlPSAT1* gene. Instead, the possibility that the reduced expression of *SlASAT1* in the *slpsat1* mutant seeds might contribute to the strong reduction of SE levels cannot be excluded.

### Generation and sterol profiling of tomato *slpsat1* x *slasat1* double knock-out mutant

The highly contrasting profiles of FS and SE in the *slpsat1* and *slasat1* single knock-out mutants prompted us to generate a *slpsat1* x *slasat1* double knock-out mutant, in order to assess the impact of simultaneous loss of function of both enzymes involved in SE biosynthesis. To this end, we crossed *slpsat31* and *slasat34* single mutants to obtain a tomato line carrying both mutant alleles in homozygosis. Sterol profiling in the double mutant revealed a metabolic phenotype very similar to that of the *slpsat1* mutants, though there was an additional reduction of total SE levels compared to the single *slpsat1* mutants, which were further depleted to 5% and 7% the content in WT seeds (**Figure 3A** and **Supplementary Table 2**) and leaves (**Figure 3B** and **Supplementary Table 3**), respectively, while FS levels were restored to WT levels in both cases (**Figures 3C, D**, and **Supplementary Tables 2** and **3**). These observations confirmed the predominance of SlPSAT1 over SlASAT1 in tomato SE biosynthesis. Interestingly, the effects of the simultaneous inactivation of *SlPSAT1* and *SlASAT1* on individual sterol species EF were disparate, since the β-sitosterol EF in leaves and cholesterol and stigmasterol in seeds were increased compared to those in corresponding organs of the *slpsat1* single mutants, in contrast to the lower values of other sterol species (**Table 1**). Taken together, the results of the sterol profiling in the single and double mutants support the view that different regulatory mechanisms operate in tomato seeds and leaves and also in the different branches of the sterol biosynthesis pathway leading to the major sterols, to maintain FS homeostasis.

### Effect of *SlPSAT1* and *SlASAT1* inactivation on the lipid droplet abundance in tomato leaves

**Figure 5:**
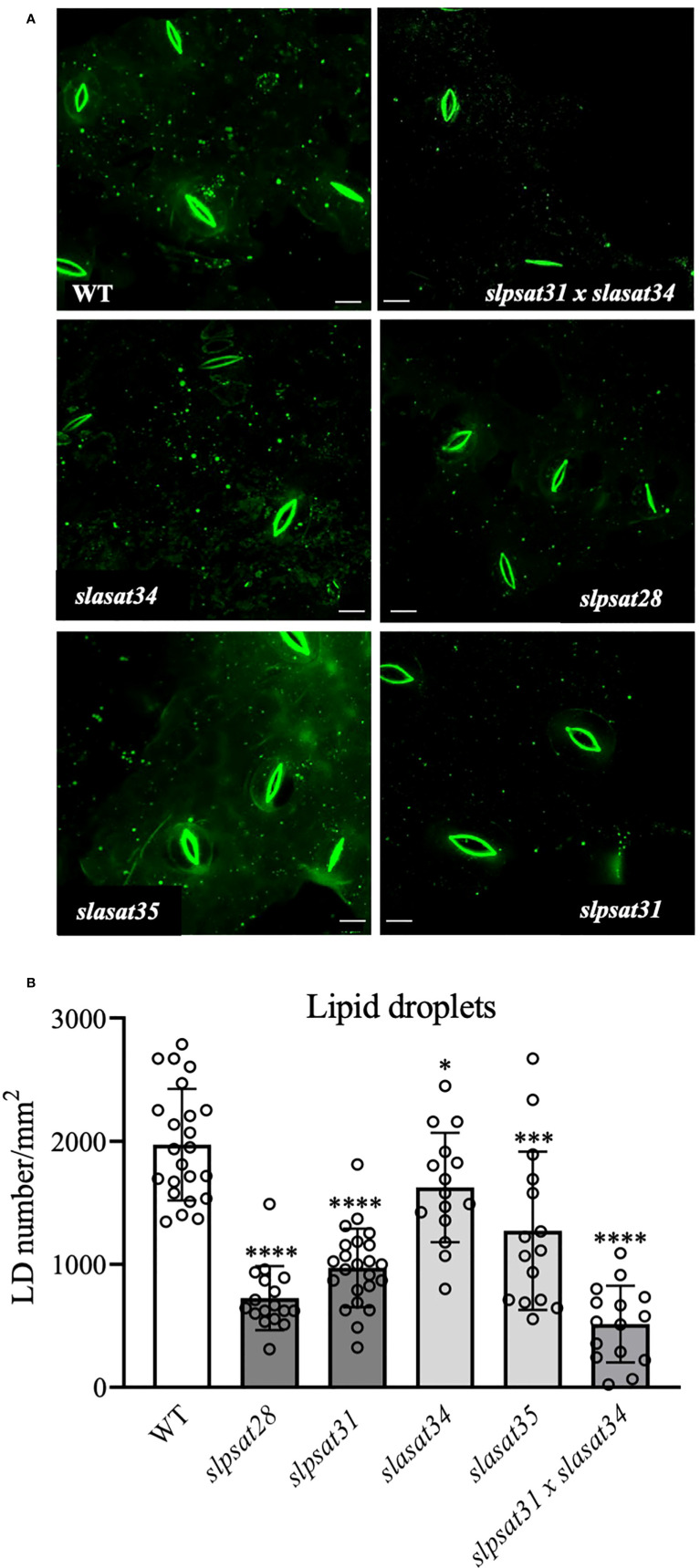
Intracellular lipid droplet distribution in leaf epidermal cells of *slpsat1*, *slasat1*, and *slpsat1* x *slasat1* knock-out mutants. **(A)** Representative confocal scanning fluorescence microscopy images showing the distribution of lipid droplets stained with TopFluor^®^ cholesterol. **(B)** Quantification of lipid droplet abundance using ImageJ 1.48v software averaged by leaf area. Data are presented as mean ± SD from three biological replicates per genotype, with n=15-23 images per genotype analyzed. Asterisks indicate values that are significantly different compared to those in the control leaves determined by t-test (* P ≤ 0.05; ***P ≤ 0.005: ****P ≤ 0.001). Scale bars = 10 μM.

To investigate the possible correlation between SE levels and the abundance of cytosolic LD, discs from the fourth leaf of WT and mutant plants were stained with TopFluor^®^ cholesterol (Gocze and Freeman, 1994) and observed with confocal microscopy. As displayed in **Figure 5A**, leaves of *slpsat1* and *slpsat1* x *slasat1* mutants contain less LDs than the WT and the *slasat1* single mutants. This is in agreement with the results of the corresponding quantitative analysis of stained LDs, which revealed reductions of ~63, ~51 and ~74% in the amount of LDs in leaves of *slpsat28*, *slpsat31* and *slpsat1* x *slasat1* mutants, respectively, compared to the WT leaves (**Figure 5B**). Unexpectedly, the abundance of LDs in both *slasat1* mutants was also lower that in the WT plants, with reductions of ~18% (*slasat34*) and ~35% (*slasat35*) (**Figure 5B**) despite the leaves of these plants accumulate much higher levels of SE than the WT (**Figure 3B**). Thus, the direct correlation between ES levels (**Figure 3B**) and LD abundance (**Figure 5B**) observed in leaves of the *slpsat1* and *slpsat1* x *slasat1* mutants does not seem to exist in the *slasat1* leaves.

### *Slpsat1* and *slpsat1 x slasat1* knock-out mutants exhibit reduced size an early leaf senescence phenotype

**Figure 6:**
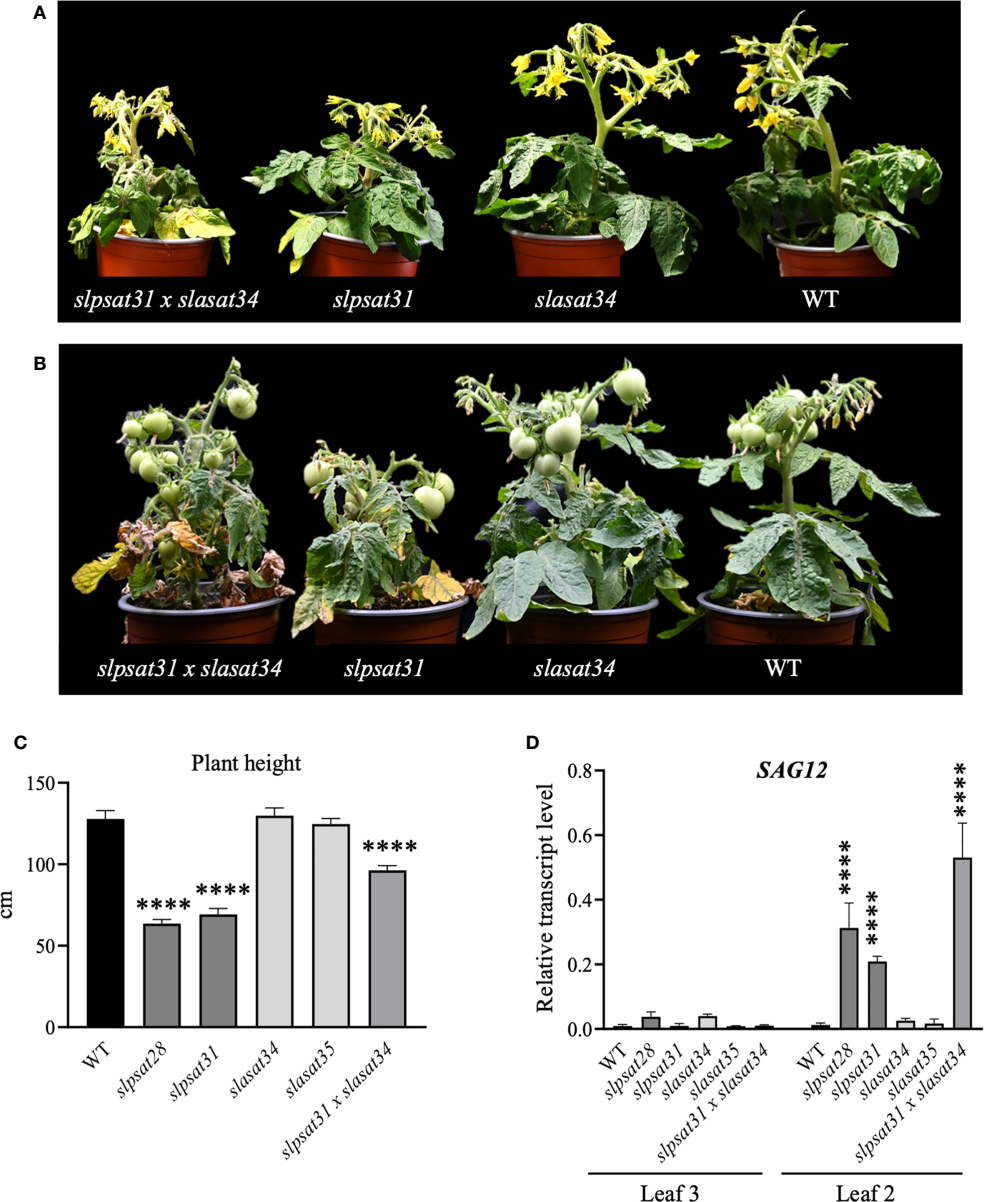
Phenotypic characterization of *slpsat1*, *slasat1*, and *slpsat1* x *slasat1* mutant plants. Representative images of 7-week-old **(A)** and 10-week-old **(B)** *slpsat1*, *slasat1*, and *slasat1* x *slpsat1* knock-out mutants grown under greenhouse conditions. **(C)** Quantitative analysis of plant height to first inflorescence of 10-week-old plants. **(D)** *SlSAG12* mRNA levels measured by RT-qPCR in RNA samples from the 2nd and 3rd leaves of 7-week-old plants. The mRNA levels of the *actin* gene were used to normalize *SlSAG12* transcript levels. Data are presented as mean ± SEM from three biological replicates per genotype with three technical replicates each (n=9). Asterisks indicate values that are significantly different compared to those in the WT plants determined by t-test (****P ≤ 0.001).

**Figure 7:**
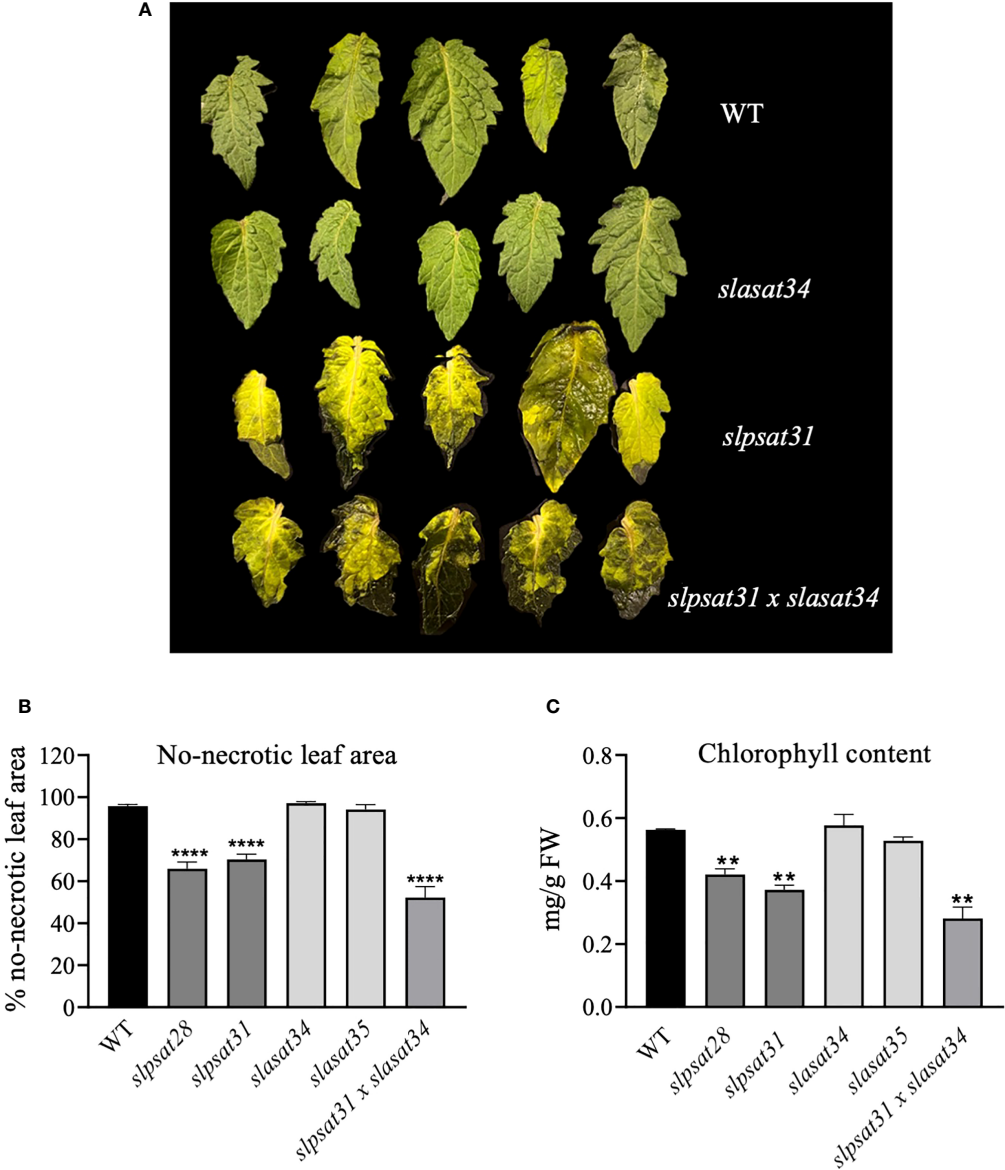
Induced senescence assay of *slpsat1*, *slasat1*, and *slpsat1* x *slasat1* detached leaves. **(A)** Representative image showing leaves detached from the indicated plants after 10 days of incubation at 24°C on water, in total darkness. The leaves of *slpsat28* and *slasat35* mutants, which are not shown in this figure, presented the same phenotype as those detached from *slpsat31* and *slasat35* mutants, respectively. **(B)** The percentage of non-necrotic leaf area was calculated by dividing the non-necrotic area by the total leaf area, which were quantified using the ImageJ2 software. **(C)** Chlorophyll content was determined in the leaves after incubation for ten days under the conditions described above. Asterisks indicate values that are significantly different compared to those in the WT plants determined by the t-test (**P < 0.01; ****P < 0.001).

Tomato CRISPR-Cas9 mutant lines described in this work were examined for morphological phenotypes associated with the loss of function of SlPSAT1, SlASAT1 or both. Seven- and ten-week-old *slasat1* mutant plants grew almost identically than WT plants (**Figures 6A-C**), whereas *slpsat1* plants were approximately 50% shorter than WT plants (**Figures 6A-C**), and a 25% reduction in height was observed in the double mutant *slpsat1 x slasat1* compared to WT (**Figures 6A-C**). These results demonstrated that a functional SlPSAT1 is essential for normal tomato plant growth while, at least under normal growth conditions, the lack of functional SlASAT1 has no evident effects on plant growth. Interestingly, the loss of function of SlASAT1 partially reverses the dwarf phenotype caused by SlPSAT1 inactivation, since a compensatory size effect was observed in the *slpsat1 x slasat1* double mutant compared with the single *slpsat1* mutants (**Figure 6C**). On the other hand, when mutant plants were about 2.5-month-old, the oldest (lower) leaves of *slpsat1* mutants started to yellow, while those of the *slpsat1 x slasat1* double mutant turned brown and dried out as typical senescent leaves. In contrast, all leaves in the *slasat1* mutants, like those of WT plants, were still green (**Figure 6B**). These observations indicated that a decrease of SE content in tomato leaves was associated to an early senescence phenotype, which was more pronounced the lower the SE content was (compare **Figures 3B** and **6B**). The positive correlation between reduced levels of SE and premature leaf senescence was confirmed by measuring the expression of the senescence marker gene *SAG12* (Noh and Amasino, 1999). To this end, total RNA was extracted from leaves of two different ages (second and third leaves) harvested from seven-week-old WT and mutant plants. At this developmental stage the third leaves of the different mutants and the WT plants were completely green, whereas the second leaves of *slpsat1* mutants showed senescence symptoms, which were more pronounced in the case of the *slpsat1 x slasat1* double mutant (**Figure 6B**). As expected, Similar basal levels of the *SlSAG12* mRNA were detected in the third leaves of WT and the mutant plants (**Figure 6D**). However, the expression of this senescence-marker gene was drastically induced in the second leaves of both *slpsat1* mutants compared to the equivalent leaves of WT plants (about 28- and 16-fold higher in *slpsat28* and *slpsat31*, respectively). Interestingly, the induction of *SlSAG12* expression was more pronounced in the *slpsat1* x *slasat1* double mutant than in the *slpsat1* single mutants (34-fold compared to WT). On the contrary, no significant differences in the *SlSAG12* transcript levels were observed in the second leaves of both *slasat1* mutants compared to the WT leaves (**Figure 6D**). To investigate if the early senescence phenotype was simply due to some developmental alteration affecting leaf ontogeny (i.e. natural senescence) or it could involve some other regulatory process, an induced leaf senescence assay was performed. Leaflets of equivalent fully green leaves detached from WT and the mutant plants (fourth leaf of 7-week-old plants) were incubated in water in permanent darkness. The leaflets from WT and *slasat1* mutant leaves remained completely green after incubation for ten days, while clear senescence symptoms (yellow and necrotic areas) were observed on leaflets from *slpsat1* and *slpsat1 x slasat1* mutant leaves (**Figure 7A**). In fact, *slasat1* mutant leaflets showed the same percentage of non-necrotic area (~97% in *slasat34* and ~94% in *slasat35*) than the WT (~96%), while this percentage decreased significantly in *slpsat1* mutants (~66% in *slpsat28* and ~70% in *slpsat31*) and was even lower in the double mutant *slpsat1 x slasat1* (~52%) (**Figure 7B**). These results were fully supported by the chlorophyll levels measured in the corresponding leaflet samples. Indeed, no differences in chlorophyll content were observed between *slasat1* and WT plants (0.59 and 0.57 mg/g FW in *slasat34* and *slasat35*, respectively, compared to 0.58 mg/g FW in WT), while this pigment content was significantly lower in the *slpsat1* mutants (0.40 and 0.37 mg/g FW in *slpsat28* and *slpsat31*, respectively) than in WT. In the double mutant the content was only about 0.30 mg/g FW (**Figure 7C**). Taken together, all the results above demonstrated that WT levels of SlPSAT1, in contrast to SlASAT1, are required for normal leaf senescence progression, even though in the absence of a functional SlPSAT1, the loss of function of SlASAT1 has a synergistic effect in the premature induction of this physiological process.

### Tomato *slpsat1* and *slasat1* mutations increase the germination speed

**Figure 8:**
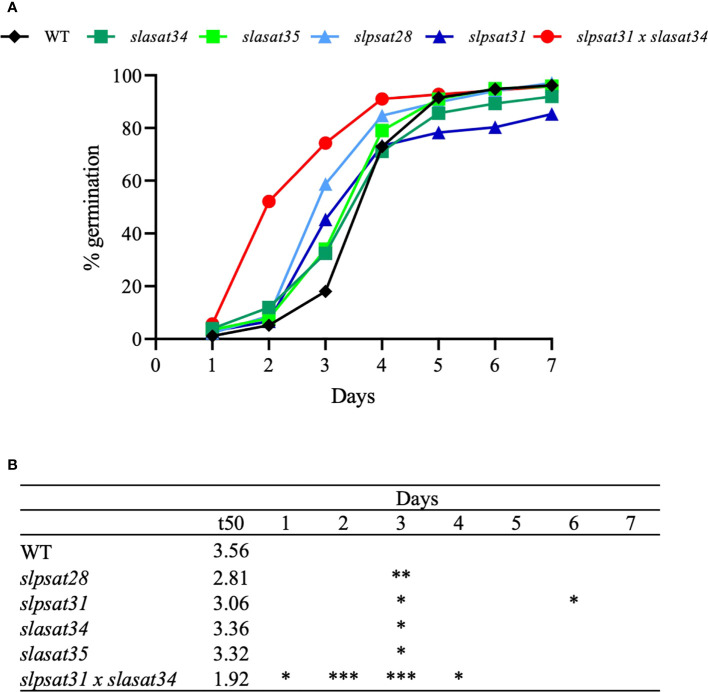
Germination rate of seeds from *slpsat1*, *slasat1*, and *slpsat1* x *slasat1* knock-out mutants. Sterilized seeds (n=60 per genotype) were sown on MS plates and allowed to germinate under long day conditions (16h light/8h dark) at 24°C. The percentage of germinated seeds at the indicated time-points **(A)** was calculated by dividing the number of seeds showing radicle emergence by the total number of seeds. Data are presented as mean ± SEM from n=4-5 replicates per genotype. The t50 values **(B)** were calculated using the formula of Coolbear et al. (1984). Asterisks indicate values that are significantly different compared to those in the control plants determined by t-test (*P < 0.05; **P < 0.01; ***P < 0.005).

It is generally accepted that mature seeds accumulate SE to meet the rapidly increasing demand for sterols when they germinate and during the early stages of seedling growth (Ferrer et al., 2017). Since loss of function of SlPSAT1 and SlASAT1 leads to altered levels of SE in seeds, being particularly remarkable the strong depletion in *slpsat1* and *slpsat1* x *slasat1* mutants (**Figure 3A** and **Table 1**), we monitored the effects of SlPSAT1 and SlASAT1 loss of function on the rate and speed of germination. As displayed in **Figure 8**, the double mutant *slpsat1 x slasat1* seeds germinated significantly faster than those from WT and the single mutants *slpsat1* and *slasat1*, whose seeds germinated also faster than WT (**Figure 8**). Thus, two days after seed imbibition, the germination percentage of the double mutant was about 10-fold higher than the WT. However, these differences decreased to 4-fold at day 3, when a significant increase of about 3-fold was also observed in the germination percentage of the single *slpsat1* mutant seeds and, to a lower extent (about 2-fold), in that of the *slasat1* seeds (**Figure 8A**). These differences were also reflected in the calculated t50 values, which were only 1.92 days for *slpsat1 x slasat1* mutant seeds compared to 3.56 days for WT seeds. A slight decrease of this value was also observed in the single mutants compared to WT (**Figure 8B**). It is worth to mention that at the end of the experimental process (7 days), no significant differences were detected between the germination percentage values of WT seeds and those from the different mutant genotypes (**Figure 8A**). All these data demonstrated that changes in SE content have no effect on tomato seeds final germination rate but cause an early germination phenotype observed not only when the SE levels are decreased, but also, although more slightly, when they are increased. This unexpected result was confirmed by the stronger early germination phenotype shown by *slpsat1* x *slasat1* seeds, which moreover demonstrated a synergistic effect of the two mutations (**Figure 8**).

## Discussion

The information currently available on plant SE biosynthesis and the sterol acyltransferases responsible for their formation is still limited. Hence, to expand the knowledge about this metabolic process and its biological significance, we generated and characterized tomato (cv. Micro-Tom) CRISPR/Cas9-induced single *slpsat1* and *slasat1* loss-of-function mutants (**Figures 1**, **2**) as well as the corresponding *slpsat1* x *slasat1* double mutant, which was obtained by crossing selected single mutants. Analysis of the total SE contents in *slpsat28*, *slpsat31*, *slasat34* and *slasat35* mutants demonstrated that SE formation in seeds and leaves depends almost solely on SlPSAT1 activity, since depletion of SE levels was observed only in the *slpsat1* mutants while SE levels in the *slasat1* mutants were increased instead of being depleted (**Figure 3**, **Table 1**). These results, along with the observation that the total amount of SE, as well as the total EF, in the *slpsat1* x *slasat1* double mutant were even lower than in the *slpsat1* single mutants (**Figure 3**, **Table 1**), strongly suggests that SlASAT1-dependent sterol esterification plays a regulatory role in tomato FS biosynthesis, as previously suggested in Arabidopsis (Chen et al., 2007). It has been proposed that ASAT1 regulates the flux through the post-squalene segment of the sterol pathway, and therefore the amount of major sterol end products, by modulating the quantity of cycloartenol (Chen et al., 2007; Zhou et al., 2019), which is the first cyclic intermediate in the plant sterol pathway (Ohyama et al., 2009). A detailed analysis of the SE profiling data in seeds and leaves of the mutant plants revealed that the contents of all measured sterol species were enhanced in the *slasat1* mutants and depleted in both the *slpsat1* and the *slpsat1* x *slasat1* mutants (**Table 1**, **Supplementary Tables 2** and **3**), thus indicating that tomato PSAT1, like its Arabidopsis counterpart (Banas et al., 2005), has a broad sterol substrate specificity.

The qualitative impact of SlPSAT1 and SlASAT1 inactivation on the profile of total SE in seeds and leaves was almost identical, although important quantitative differences were detected. The relative effect of SlPSAT1 inactivation on total SE levels was more pronounced in seeds than in leaves (~90% *vs* ~70% reduction, respectively, compared to WT), while that of SlASAT1 inactivation in seeds was far smaller than in leaves (~50% *vs* ~300% increase, respectively, compared to WT) (**Figure 3**). However, normalization of SE levels to the FS levels to calculate the total EF, revealed that the effect of SlPSAT1 inactivation was indeed more pronounced in seeds than in leaves (~8-fold *vs* ~3.5-fold reduction, respectively, compared to WT) and, conversely that SlASAT1 inactivation actually had a very similar effect in both organs (~1.5-fold increase compared to WT) (**Table 1**). A differential response was also observed in the seeds and leaves of the double mutant (~12-fold *vs* ~8-fold reduction, respectively, compared to WT). Furthermore, this analysis unveiled a contrasting impact on the EF of specific sterol species, such as cholesterol in seeds and β-sitosterol in leaves of the double mutant, compared to the total EF and that of other individual sterols. It is also worth noting that the stigmasterol EF was particularly sensitive to SlASAT1 inactivation compared to the EF of the remaining sterols (**Table 1**). These observations indicate that the regulatory mechanisms controlling SE-dependent FS homeostasis are different not only in seeds and leaves but also in the different branches of the sterol biosynthesis pathway leading to the main sterol end products. This cannot be considered completely unexpected taking into account the distinct roles of sterols in these two organs and the differences in the biological properties of 24-ethylsterols (stigmasterol and β-sitosterol), 24-methylsterols (campesterol) and sterols with no alkyl substituent at this position (cholesterol).

It is also worth noting that the increased amounts of ES found in seeds and leaves of the *slasat1* mutants compared to their WT counterparts cannot be attributed to *SlPSAT1* transcriptional upregulation, since its mRNA levels remained either unaltered in seeds (**Figure 4A**) or even diminished in leaves (**Figure 4B**). Thus, it remains to be established whether the observed accumulation of SE levels in the *slasat1* mutant seeds and leaves is due to post-transcriptional upregulation of the SlPSAT1 enzyme activity, simply to a greater availability of the FS substrates of PSAT1, or both. In fact, it has been proposed that PSAT1 is not rate-limiting for SE formation in Arabidopsis (Noiriel et al., 2004; Bouvier-Navé et al., 2010) and the tea tree (Zhou et al., 2019). On the other hand, the possibility that the enhanced levels of SE in the *slasat1* mutants could result from the upregulation of some of the seven additional genes in the tomato genome coding for SlASAT-like proteins (Lara et al. 2018) cannot be completely ruled out, although the observation that the levels of SE in leaves and seeds of the double *slpsat1 x slasat1* mutant are lower than those in the single *slpsat1* mutants (**Figures 3A, B**) argues against this hypothesis.

Interestingly, the inactivation of SlPSAT1 and SlASAT1 also led to changes in the levels of FS, albeit less pronounced than those of SE, which also differ depending on the organ and the mutant genotype. While in the leaves there was a moderate but significant increase of FS contents in both mutants, the content of FS in seeds remained unchanged in the *slpsat1* mutants and was significantly reduced in the *slasat1* mutants (**Figure 3**). These organ-dependent and highly contrasting metabolic responses to SlPSAT1 and SlASAT1 inactivation likely reflect the differences in the physiological role and the regulatory mechanisms controlling SE levels in these organs. Seeds contain about 5 times more SE than leaves (**Figure 3** and **Supplementary Tables 2**and **3**), and their suggested primary role is to serve as a storage pool of sterols that can be easily mobilized to meet the increasing demand of plasma membrane components during the early stages of seedling growth. In the leaves, SE are also suggested to play a role in providing FS to support rapid expansion (Zhou et al., 2019), in addition to their involvement in maintaining sterol homeostasis in the cell membranes during growth and development, including the recycling of membrane sterols as senescence progresses or during stress (Bouvier-Navé et al., 2010; Kumar et al., 2018). Lastly, it is also worth to mention that residual levels of SE were still detected in the *slpsat1* x *slasat1* double knock mutant, suggesting that other acyltransferases may contribute to SE formation in both seeds and leaves. In fact, as mentioned above, the tomato genome contains up to seven genes coding for ASAT-like proteins (Lara et al., 2019).

Despite a number of metabolic responses in tomato plants lacking functional SlPSAT1 or SlASAT1 are similar to those previously reported in Arabidopsis, there are also some relevant differences. The dramatic increase of SE in leaves of the *slasat1* tomato mutants (**Figure 3B**) was not observed in the rosette leaves of the Arabidopsis *asat1-1* mutant, whose SE levels are even lower than those in the control leaves (Bouvier-Navé et al., 2010; Lara et al. 2018). Similarly, the increase of SE levels observed in tomato *slasat1* seeds (**Figure 3A**) is also in contrast to the unchanged content of SE in Arabidopsis seeds (Bouvier-Navé et al., 2010). Remarkable differences were also observed in the effects on plant growth and important developmental processes such as leaf senescence and seed germination. A functional SlPSAT1, but not SlASAT1, is necessary for proper tomato plant growth (**Figures 6A, B**), which is consistent with the suggested role of SE as a source of sterols to sustain rapid expansion of vegetative organs (Zhou et al., 2019). The reason why a similar phenotype was not observed in the Arabidopsis mutants lacking a functional PSAT1 (Bouvier-Navé et al., 2010) is unclear, although it might be speculated that it could be due, at least in part, to the relative lower levels of SE in the vegetative organs of tomato *slpsat1* and *slpsat1 x slasat1* mutants (**Figure 3B**) compared to those in the equivalent Arabidopsis mutants (Bouvier-Navé et al., 2010), so that the necessity of a minimum SE threshold level for proper plant growth cannot be excluded.

The involvement of SE in tomato senescence regulation, as previously reported in Arabidopsis (Bouvier-Navé et al., 2010), would be mainly mediated by SlPSAT1, since an early senescence phenotype was observed in both *slpsat1* mutant lines, but not in the *slasat1* ones (**Figures 6A** and **7A**). Interestingly, in contrast to that observed in Arabidopsis, where the *asatpsat* mutants showed the same early leaf senescence phenotype than the single *psat1* mutants (Bouvier-Navé et al., 2010), a synergistic effect of *slpsat1* and *slasat1* mutations was observed in tomato, since the senescence phenotype of the tomato double mutant *slpsat1* x *slasat1* was clearly stronger than that of *slpsat1* (**Figures 6A** and **7A**). This observation is fully consistent with the expression profile of the senescence-marker gene *SlSAG12* detected in leaves of different ages from WT and mutant plants (**Figure 6D**). Moreover, the senescence-promoting effect of the *slpsat1* mutation and the synergistic effect of both mutations were also observed in an induced senescence assay using detached leaves (**Figure 7**). In aging tissues, sterols and fatty acids released from disorganized membranes would be converted to SE (Holmer et al., 1973; Chen et al., 2007). This explains the increase of SE content observed during senescence in several plant species and organs, including leaves (Dyas and Goad, 1993; Mckegney et al., 1995; Bouvier-Navé et al., 2010; Li et al., 2016). Thus, the impaired capacity of *slpsat1 and slpsat1 x slasat1* mutants to esterify FS in leaves (**Figure 3B**) is likely to be one of the reasons underlying the early senescence phenotype observed in the leaves of these mutants which, on the other hand, is not detected in the *slasat1* mutants (**Figures 6** and **7**) that are able to produce and accumulate high levels of SE (**Figure 3B**). As mentioned previously, SE accumulate in cytosolic LDs, whose primary role in leaves seems to be the removal of potentially toxic FS and other lipid catabolites scavenged from damaged membranes during stress or senescence (Huang, 2018). The reduced amounts of LDs detected in leaves of tomato *slpsat1*, *and slpsat1 x slasat1* mutant plants compared to WT (**Figure 5**) are in close correlation with the temporal senescence differences observed between these genotypes and the WT. Thus, it is reasonable to assume that the reduced abundance of LDs in these mutants reflects the impaired capacity to esterify sterols which, in turn, prevents proper maintenance of FS membrane homeostasis and accelerates the onset of senescence.

A number of studies support the notion that LD formation is strongly associated with increasing levels of SE (Maillot-Vernier et al., 1991; Gondet et al., 1994; Bouvier-Navé et al., 2010; Shimada et al., 2019). However, such positive correlation between SE overaccumulation and enhanced LD abundance was not observed in the case of the *slasat1* leaves, in which the number of LD was lower than in WT leaves (**Figure 5**) despite their much higher levels of ES (**Figure 3B**). In this respect, it is interesting to note that a certain lack of correlation between LD abundance and SE contents has also been described during the diurnal cycle in tea leaves (Zhou et al., 2019). Either way, the enhanced capacity of *slasat1* leaves to form SE might explain why these mutants do not show any symptom of premature senescence, but at the same time raises the question about the reason underlying the unexpected reduced accumulation of LDs. It has been recently suggested that altered membrane sterol composition may compromise endoplasmic reticulum (ER)-based LD biogenesis in Arabidopsis seeds and leaves due to the capacity of sterols to modulate cell membranes physicochemical properties and the organization of putative PSAT1-localized ER microdomains where ES would be formed and segregated into LDs (Yu et al., 2021). Thus, it might be speculated that the altered profile of FS in leaves of the *slasat1* mutants (**Figure 3D** and **Supplementary Table 3**) may hamper proper formation of LDs in their leaves through a similar mechanism. Also, the possibility that SE content/concentration in the LDs of *slasat1* mutants is higher compared to that in LDs of WT leaves to accommodate excess SE without increasing the number of LDs cannot be either excluded.

The rate of seed germination has been correlated with a proper seed reserve content (Sun et al., 2015; Shrestha et al., 2016; Zhao et al., 2018). This physiological process involves the breakdown of storage reserves accumulated in the endosperm, like carbohydrates, lipids and proteins, into soluble molecules that are mobilized through the embryo to allow seedling development (Bewley and Black, 1994; Pritchard et al., 2002). Previous studies indicate that changes in seed sterol levels affect seed germination (Du et al., 2022) and it is generally believed that SE are important to supply the necessary FS to sustain seed germination and seedling growth during the early development stages (Dyas and Goad, 1993; Harker et al., 2003). In this work we demonstrate that changes in tomato seeds SE contents, either a moderate increase or a strong reduction (**Figure 3A**), do not affect significantly the germination rate, as evidenced by the similar percentage of WT and the different mutant seeds germinated after 7 days (**Figure 8**). On the contrary, the *slpsat1 x slasat1* mutant seeds, which are almost completely devoid of SE, germinated significantly faster than the single *slpsat1* and *slasat1* mutant seeds, whose germination speed was slightly higher than that of WT seeds (**Figure 8**). The difference in germination speed between *slpsat1* x *slasat1* and the *slpsat1* seeds is intriguing given the small differences in their SE content (**Figure 3A** and **Supplementary Table 2**), although the possibility that SE levels in the *slpsat1* x *slasat1* seeds are below a given threshold necessary for normal germination cannot be excluded. In any case, this different germination behavior cannot be attributed to differences in FS levels (**Figure 3C** and **Supplementary Table 2**) and, moreover, reinforces the synergistic effect of both mutations previously observed in the early senescence phenotype (**Figures 6**–**8**).

Sterol mobilization during seed germination has been reported in several plant species, including tomato (Duperon et al., 1984; Garg and Nes, 1985; Huang and Grunwald, 1988; Guo et al., 1995; Zhang et al., 2007). The early germination phenotype of *slpsat1* x *slasat1* seeds could result from a better reserve mobilization rather than from changes in its content, which, as mentioned above, is primarily related with the germination rate. Moreover, germination is inversely proportional to a weaker endosperm and seed coat (Steinbrecher and Leubner-Metzger, 2017). It is thus tempting to hypothesize that early seed germination in tomato SE biosynthetic mutants could be due to the potential effect of the altered FS and ES profiles in the seed coat formation. Sterols might also affect directly the germination signaling pathway, since during plant growth and development phytosterols can interact with different hormone pathways (Vriese et al., 2021), including gibberellins (He et al., 2003), which have been reported as essential factors for tomato seed germination (Groot and Karssen, 1987; Bassel et al., 2004). Work is currently in progress to shed light on the biochemical, molecular and physiological mechanisms underlying the tomato phenotypes associated to altered profiles of SE.

## Data availability statement

The original contributions presented in the study are included in the article/**Supplementary Material**. Further inquiries can be directed to the corresponding authors.

## Author contributions

AB-M and JL-T performed most of the experimental work. NL performed the germination assay. NL and CD contributed to the analysis of senescence parameters. TA and AF conceived the research, managed and supervised the project, and wrote the manuscript. All the authors analyzed data. All authors contributed to the article and approved the submitted version.

## Funding

This work was supported by grants AGL2017-88842-R and PID2021-126591OB-I100 funded by MCIN/AEI/10.13039/501100011033, and by FEDER, a way of making Europe (Spain). This work was also supported by grants CEX2019-000902-S funded by MCIN/AEI/10.13039/501100011033 (Spain) and by the CERCA Programme/Generalitat de Catalunya.

## Acknowledgments

JL-T received a doctoral fellowship (Ref. PRE2018-084652) from the Ministerio de Ciencia e Innovación (Spain). NL has financial support from the European Union’s Horizon 2020 research and innovation program under the Marie Skłodowska-Curie grant agreement No 945043. CD received a Ph.D. fellowship (CSC202109110067) from the China Scholarship Council (China). We also thank Pilar Fontanet for her invaluable help in the generation of the tomato transgenic lines characterized in this study, and the CRAG greenhouse staff members for the maintenance of plants.

## Conflict of interest

The authors declare that the research was conducted in the absence of any commercial or financial relationships that could be construed as a potential conflict of interest.

## Publisher’s note

All claims expressed in this article are solely those of the authors and do not necessarily represent those of their affiliated organizations, or those of the publisher, the editors and the reviewers. Any product that may be evaluated in this article, or claim that may be made by its manufacturer, is not guaranteed or endorsed by the publisher.
