# Supplementary material for: Effects of impaired steryl ester biosynthesis on tomato growth and developmental processes
Source: Front Plant Sci. 2022 Sep 29;13:984100. doi: 10.3389/fpls.2022.984100 (PMC9557751; doi:10.3389/fpls.2022.984100)
Supplement: Supplementary file 1 [file Table_1.doc]

Supplementary Table 1

**Table S1:** Primers used in this work.

| **Primer name** | **Primer sequence** | **Application** |
| --- | --- | --- |
| ASAT-1 CRISPR Fw | ATTG**GGCTTGGCCCGCGGGCTTCT** | DNA heteroduplex encoding sgRNAs. Complementary sequences in each primer pair are shown in bold |
| ASAT-1 CRISPR Rv | AAAC**AGAAGCCCGCGGGCCAAGCC** |
| PSAT-1 CRISPR Fw | ATTG**GGCGATTACTCGAAGCTGTC** |
| PSAT-1 CRISPR Rv | AAAC**GACAGCTTCGAGTAATCGCC** |
| AtU6-26 prom Fw | ACAGTCTTTCACCTCTCTTTGG | Genotyping of kanamycin-resistant primary transformants (T0). |
| AtU6-26 prom Rv | GGCCTGCTTCTCTTCTTTCA |
| sgRNA ASAT1 Fw | GCAAAGTCCAACAAAGGTCAAAAG | Amplification of genomic fragments encompassing sgRNA target sequences in *SlASAT1* and *SlPSAT1* genes. |
| sgRNA ASAT1 Rv | TATTCGCGGAGAGGCTTAGA |
| sgRNA PSAT1 Fw | CCAATTCCCAGCGGCAAAAAGTC |
| sgRNA PSAT1 Rv | AGTGTCGAGCCAGACTAAATCG |
| SAG12 Fw | CACACCCTAAGATTATGTCCTCCTC | Determination of SAG12 mRNA levels in leaves by qRT-PCR |
| SAG12 Rv | CATCCACATTCCATTTGGTCCTTGA |
| SlPSAT1-fw | TGCTATTGGGATTACGGGAAAG | Determination of SlPSAT1 mRNA levels in leaves by RT-qPCR. |
| SlPSAT1-qP-rev | GTGTGACAGGATGTGAGATGTAG |
| SlASAT1-fw | GAAGTTAGTTGAAAATGGTTCTAGTG | Determination of SlASAT1 mRNA levels in leaves by RT-qPCR. |
| SlASAT1-rev | TTGACCTTTGTTGGACTTTGC |
| Actin Fw | CCTTCCACATGCCATTCTCC | Reference gene for RT-qPCR expression analysis. |
| Actin Rv | CCACGCTCGGTCAGGATCT |
