# Supplementary material for: Effects of impaired steryl ester biosynthesis on tomato growth and developmental processes
Source: Front Plant Sci. 2022 Sep 29;13:984100. doi: 10.3389/fpls.2022.984100 (PMC9557751; doi:10.3389/fpls.2022.984100)
Supplement: Supplementary file 2 [file Table_2.docx]

Supplementary Table 2

**Table S2**. **FS and SE quantification in seeds of wt, *slasat1*, *slpsat1* and *slpsat1* x *slasat1* mutant**s. Data are shown as average values from three biological replicates with SEM in parentheses. n.d. stands for not detected. Significant changes compared to wild-type (WT) seeds are indicated by asterisks (*P<0.05; **P<0.01; ***P<0.005).

| **Seeds** | **WT** | ***slasat34*** | ***slasat35*** | ***slpsat28*** | ***slpsat31*** | ***slpsat31*** x ***slasat34*** |
| --- | --- | --- | --- | --- | --- | --- |
|  | **(μg/mg dry weight)** | | | | | |
| **Total FS** | **1.3710** (0.0090) | **0.7923*** (0.0761) | **0.8711****  (0.0277) | **1.3121** (0.0270) | **1.5726** (0.0927) | **1.3689**  (0.0079) |
| Cholesterol | 0.0812 (0.0053) | 0.0410 (0.0043) | 0.0473* (0.0017) | 0.0353** (0.0011) | 0.0328* (0.0012) | 0.0175**  (0.0016) |
| Campesterol | 0.0651 (0.0026) | 0.0272* (0.0028) | 0.0417* (0.0007) | 0.0423** (0.0017) | 0.0477 (0.0031) | 0.0382***  (0.0014) |
| Stigmasterol | 0.2008 (0.0098) | 0.0959* (0.0109) | 0.0833* (0.0058) | 0.1481* (0.0025) | 0.1471 (0.0049) | 0.1938  (0.0030) |
| Sitosterol | 0.9473 (0.0123) | 0.5914* (0.0499) | 0.6650*** (0.0201) | 1.0558 (0.0209) | 1.2509* (0.0752) | 1.0571*  (0.0135) |
| Isofucosterol | 0.0766  (0.0055) | 0.0368  (0.0088) | 0.0338*  (0.0007) | 0.0306* (0.0009) | 0.0941 (0.0101) | 0.0623  (0.0024) |
|  | **WT** | ***slasat34*** | ***slasat35*** | ***slpsat28*** | ***slpsat31*** | ***slpsat31*** x ***slasat34*** |
|  | **(μg/mg dry weight)** | | | | | |
| **Total SE** | **0.8193** (0.0776) | **1.2154**** (0.0178) | **1.3188***** (0.0161) | **0.0733**** (0.0054) | **0.0673**** (0.0155) | **0.0429****  (0.0101) |
| Cholesteryl | 0.2617 (0.0182) | 0.3663* (0.0047) | 0.3171 (0.0104) | 0.0044*** (0.0004) | n.d. | 0.0035**  (0.0003) |
| Campesteryl | 0.0439 (0.0019) | 0.0459 (0.0021) | 0.0368* (0.0007) | n.d. | n.d. | n.d. |
| Stigmasteryl | 0.0323 (0.0037) | 0.0597 (0.0033) | 0.0303 (0.0020) | 0.0005* (0.0003) | n.d. | 0.0054*  (0.0034) |
| Sitosteryl | 0.3675 (0.0432) | 0.4947 (0.0133) | 0.7561*** (0.0343) | 0.0472* (0.0067) | 0.0673** (0.0155) | 0.0236*  (0.0058) |
| Isofucosteryl | 0.0776 (0.0122) | 0.1256*** (0.0113) | 0.1026 (0.0080) | n.d. | n.d. | n.d. |
| Cycloartenyl | 0.0364 (0.0042) | 0.1231*** (0.0054) | 0.0759* (0.0111) | 0.0212** (0.0113) | n.d. | 0.0103*  (0.0013) |
