# Supplementary material for: Effects of impaired steryl ester biosynthesis on tomato growth and developmental processes
Source: Front Plant Sci. 2022 Sep 29;13:984100. doi: 10.3389/fpls.2022.984100 (PMC9557751; doi:10.3389/fpls.2022.984100)
Supplement: Supplementary file 3 [file Table_3.docx]

Supplementary Table 3

**Table S3.** **FS and SE quantification in leaves of wt, *slasat1*, *slpsat1* and *slpsat1* x *slasat1* mutants**. Data are shown as average values from four biological replicates with SEM in parentheses. n.d. stands for not detected. Significant changes compared to wild-type leaves are indicated by asterisks (*P<0.05; **P<0.01; ***P<0.005).

| **Leaves** | **WT** | ***slasat34*** | ***slasat35*** | ***slpsat28*** | ***slpsat31*** | ***slpsat31*** x ***slasat 34*** |
| --- | --- | --- | --- | --- | --- | --- |
|  | **(μg/mg dry weight)** | | | | | |
| **Total FE** | **0.2184** (0.0094) | **0.3676***** (0.0109) | **0.2808***  (0.0050) | **0.3115***** (0.0036) | **0.3245*** (0.0202) | **0.1949**  (0.0035) |
| Cholesterol | 0.0248 (0.0015) | 0.0504* (0.0046) | 0.0408* (0.0037) | 0.0187*** (0.0008) | 0.0256 (0.0009) | 0.0336*  (0.0003) |
| Campesterol | 0.0090 (0.0006) | 0.0331* (0.0046) | 0.0291* (0.0050) | 0.0086 (0.0006) | 0.0105 (0.0008) | 0.0024***  (0.0002) |
| Stigmasterol | 0.1012 (0.0047) | 0.1060 (0.0072) | 0.0846* (0.0036) | 0.1169 (0.0045) | 0.1480* (0.0044) | 0.1082  (0.0015) |
| Sitosterol | 0.0834 (0.0034) | 0.1780** (0.0151) | 0.1263* (0.0094) | 0.1674*** (0.0027) | 0.1404* (0.0150) | 0.0507*  (0.0028) |
|  | **WT** | ***slasat34*** | ***slasat35*** | ***slpsat28*** | ***slpsat31*** | ***slpsat31*** x ***slasat 34*** |
|  | **(μg/mg dry weight)** | | | | | |
| **Total SE** | **0.1529** (0.0099) | **0.5874***** (0.0348) | **0.6233***** (0.0352) | **0.0408**** (0.0067) | **0.0443***** (0.0037) | **0.0106*****  (0.0013) |
| Cholesteryl | 0.0163 (0.0020) | 0.097*** (0.0062) | 0.0750*** (0.0022) | 0.0037** (0.0003) | 0.0035** (0.0006) | n.d. |
| Campesteryl | 0.0095 (0.0007) | 0.0360** (0.0049) | 0.0313*** (0.0021) | n.d. | n.d. | n.d. |
| Stigmasteryl | 0.0081 (0.0011) | 0.0387* (0.0052) | 0.0254* (0.0022) | n.d. | n.d. | n.d. |
| Sitosteryl | 0.0459 (0.0027) | 0.2651*** (0.0257) | 0.2330*** (0.0134) | 0.0142*** (0.0030) | 0.0157*** (0.0014) | 0.0106***  (0.0013) |
| Cycloartenyl | 0.0731 (0.0040) | 0.1506* (0.0175) | 0.2585*** (0.0221) | 0.0230* (0.0068) | 0.0251*** (0.0043) | n.d. |
